# Multicomponents of spin-spin relaxation, anisotropy of the echo decay, and nanoporous sample structure

**DOI:** 10.21203/rs.3.rs-2893081/v1

**Published:** 2023-05-10

**Authors:** Theodore Aptekarev, Gregory Furman, Vladimir Sokolovsky, Alexander Panich, Yang Xia

**Affiliations:** 1 Physics Department, Ben Gurion University of the Negev, Beer Sheva, Israel; 2 Physics Department, Oakland University, Rochester, MI, US

**Keywords:** Echo decay anisotropy, Dipole–dipole interactions, Nanopore, Nanostructure, NMR

## Abstract

We have experimentally and theoretically investigated multicomponent ^1^H nuclear magnetic resonance (NMR) echo decays in a-Si:H films containing anisotropic nanopores, in which randomly moving hydrogen molecules are entrapped. The experimental results are interpreted within the framework of the previously developed theory, in which a nanoporous material is represented as a set of nanopores containing liquid or gas, and the relaxation rate is determined by the dipole–dipole spin interaction, considering the restricted motion of molecules inside the pores. Previously, such characteristics of a nanostructure as the average volume of pores and their orientation distribution were determined from the angular dependences of the spin–spin and spin–lattice relaxation times.

We propose a new approach to the analysis of the NMR signal, the main advantage of which is the possibility of obtaining nanostructure parameters from a single decay of the echo signal. In this case, there is no need to analyze the anisotropy of the relaxation time $T_{2}$, the determination of which is a rather complicated problem in multicomponent decays. Despite multicomponent signals, the fitting parameter associated with the size and shape of nanopores is determined quite accurately. This made it possible to determine the size and shape of nanopores in a-Si:H films, herewith our estimates are in good agreement with the results obtained by other methods. The fitting of the decays also provides information about the nanostructure of the sample, such as the standard deviations of the angular distribution of pores and the polar and azimuthal angles of the average direction of the pore axes relative to the sample axis, with reasonable accuracy. The approach makes it possible to quantitatively determine the parameters of the non-spherical nanoporous structure from NMR data in a non-destructive manner.

## 1 Introduction

Nuclear magnetic resonance (NMR) and nuclear magnetic resonance imaging (MRI) are the most sensitive and powerful tools for identifying the structure of both pure compounds and mixtures as either solids or liquids [1-4]. A remarkable feature of these methods is their ability to be used for research on different levels of organization of matter, from atoms [1,2] and molecules [5] to solids [3, 4], cells [6, 7], membranes [7], and tissues of living organisms [7, 8].

The spin-spin interactions play an important role in NMR and MRI due to the fact that coupling constants characterizing the interactions depend on the structural parameters of the tested materials [1-5]. In solids, dipole-dipole interactions (DDI) of nuclear spins are often the main interactions, which are responsible for the shape of NMR spectra and spin-spin and spin-lattice relaxation processes [1-4]. This is especially true for the spins of hydrogen atoms [9] owing to the weak electron-nuclear coupling. Measurements of the relaxation times allow one to study the dynamic and structural properties of materials, including their microstructure and composition [3, 5, 8].

In recent years, NMR and MRI methods have been used to study the nanostructures of nanoporous materials, in which mobile gas or liquid molecules are trapped in pores less than a few hundred nanometers in size [10-13]. Such materials can be found commonly in nature, including biological systems and living organisms, and possess a number of unique properties. One of the main advantages of magnetic resonance methods is their non-invasiveness and non-destructiveness.

In our previous works, we studied the relaxation processes in molecules that are entrapped in nano-sized cavities (nanopores) of the materials [14-22]. We have demonstrated that the parameters of nanostructures can be extracted from the angular dependence of the relaxation times $T_{1}$, $T_{1\rho }$, and $T_{2}$. To explain in detail the anisotropy of the relaxation times for liquid and gas entrapped in nanopores that have a characteristic size up to hundreds of nanometers, the model considered the restricted random motion of molecules at averaging the spin Hamiltonian [14-22]. It was shown [16-22] that the averaged Hamiltonian which describes the dipole-dipole interactions in the nanopores can be characterized by an effective coupling constant. Averaging the spatial part of the dipolar Hamiltonian, we developed the models which describe very non-trivial spin dynamics and allow us to obtain analytical expressions for the relaxation times $T_{1}$, $T_{1\rho }$, and $T_{2}$. These expressions establish the relationship between the sample nanostructure and the anisotropy of the relaxation times. However, determining the relaxation times, especially the transverse relaxation time from experiments where the measured signal is multicomponent, is not a well-defined procedure [23-26]. Usually, a relaxation time is determined using one- or two- exponential functions for fitting experimental data [25, 26]. It was shown [14-22] that the nanostructure of nanoporous materials can be analyzed using the angular dependence of the relaxation time obtained by a simple fitting of experimental data for a sample, that is put at different angles relative to an applied magnetic field. The fitting by one- or two-exponential functions is more problematic for nanoporous samples where the measured signal is formed by a superposition of signals from a set of nanopores with different orientations, volumes, etc. [14, 24-26].

The purpose of the present study was to establish a correlation between the structural features of nanoporous materials (orientation of nanopores, their volume and shape) and the echo decays, bypassing the procedure of determination of the transverse relaxation time. The advantage of the proposed method is the approximation of experimental data by a multicomponent exponential function. The developed approach opens up a way of studying nanostructures by analyzing single echo decay.

This paper is organized as follows: in Section 2 for convenience of the readers, we briefly show that the angular dependence (anisotropy) of the transverse relaxation time results from the residual dipole-dipole interaction in liquid or gas entrapped in nanopores. In Section 3, we describe the experimental details. The method to determine the internal nanostructural parameters in hydrogenated amorphous silicon (a-Si-H) films by using the theoretical model and the experimental data is considered in Section 4. In the last section, we summarize our results.

## 2 Theory

In this section, we first describe the spin-spin relaxation in a single nanopore containing liquid or gas molecules and then consider the effect of nanopore orientation disorder in a sample on the echo decay.

### 2.1 Echo decay in a single nanopore

**Fig. 1.**
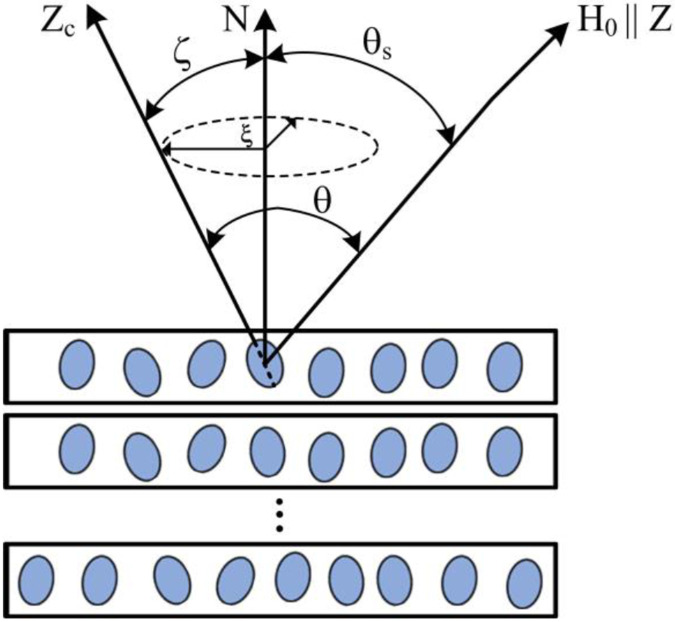
Sketch of a multilayer silicon a-Si:H film with elongated nanopores containing hydrogen molecules. $\theta_{S}$ is the angle between the external magnetic field ${\vec{H}}_{0}$ and the normal to the sample surface N-axis; θ is the angle between the external magnetic field ${\vec{H}}_{0}$ and the ZC-axis of a nanocavity; ζ and ξ are the polar and azimuthal angles characterizing the deviation of the ZC-axis of a nanopore from the **N**-axis.

For gas or liquid entrapped in a nanopore with sizes less than 1 μm the Hamiltonian of a spin system can be averaged over the random motion of molecules [14-22]. The averaged Hamiltonian describing the dipole-dipole (DD) interaction in the magnetic field ${\vec{H}}_{0}$ along the z-axis (${\vec{H}}_{0}\|z$) is given by [27, 28]:

(1) $${\hat{H}}_{d}=\frac{\gamma^{2}\hbar }{2V}\left(3\;\cos^{2}\theta -1\right)F\sum_{i<j}\left(3I_{zi}I_{zj}-{\vec{I}}_{i}{\vec{I}}_{j}\right),$$

where γ is the nuclear gyromagnetic ratio, ℏ is the reduced Planck constant, V is the nanopore volume and F is the form-factor [28], θ denotes the angle between the main $Z_{C}$-axis of the nanopore and the external magnetic field ${\vec{H}}_{0}$ (Fig. 1), $I_{zj}$ is the projections of the spin operator ${\vec{I}}_{j}$ onto the *Z* – axis of the j -th nuclear spin. Eq (1) was obtained for ellipsoidal or cylindrical nanopores. Consideration of nanopores as ellipsoids or cylinders is correct in many cases, including nanopores in a-Si:H films [21, 22, 29-33].

To detect the anisotropy of the relaxation processes, the sample is put at different angles relative to the direction of the applied magnetic field. In this case, the measure of change in orientation of the sample is the angle $\theta_{S}$ between the normal to the sample surface and the direction of the magnetic field. Therefore, to describe all possible orientations of a nanopore, it is necessary to rewrite Eq. (1), in the form accounting the sample rotation:

(2) $${\hat{H}}_{d}=\frac{\gamma^{2}\hbar }{2V}\left[3\;f^{2}(\theta_{S},\zeta ,\xi )-1\right]F\sum_{i<j}\left(3\;I_{zi}I_{zj}-{\vec{I}}_{i}{\vec{I}}_{j}\right),$$

where

(3) $$f(\theta_{S},\zeta ,\xi )=\cos\;\theta_{S}\;\cos\;\zeta -\sin\;\theta_{S}\;\sin\;\zeta \;\cos\;\xi$$

$\theta_{S}$ is the angle between the external magnetic field ${\vec{H}}_{0}$ and the normal to the sample surface N-axis (Fig. 1).

The transverse relaxation time can be calculated using its relationship with the local dipolar field [3]: $T_{2}\approx \omega_{\mathrm{loc}}^{-1}$ and a local dipole field is defined as $\omega_{\mathrm{loc}}=\sqrt{\frac{\mathrm{Tr}({\bar{H}}_{d}^{2})}{\mathrm{Tr}(I_{z})}}$. Using the expression for the averaged secular part ${\bar{H}}_{d}$ of the dipole-dipole interactions (Eq. 3) the transverse relaxation time for a single nanopore can be obtained [14-16]:

(4) $$T_{2}(\theta_{S},\zeta ,\xi )=\frac{1}{\eta |\left(3\;f^{2}(\theta_{S},\zeta ,\xi )-1\right)|}$$

where

(5) $$\eta =\frac{\eta^{2}\hbar }{4}|F|\sqrt{\frac{3\eta }{V}},$$

ρ is the spin density of liquid or gas.

According to the phenomenological Bloch equations a transverse relaxation from a single nanopore can be described by an exponential decay:

(6) $$S(t,\theta_{S},\zeta ,\xi )=M_{0}\exp\left(-\frac{t}{T_{2}(\theta_{S},\zeta ,\xi )}\right),$$

where $M_{0}$ is the initial signal intensity at t=0.

### 2.2 Echo decay in a sample with orientational distribution of nanopores

Eqs. (4), (5), and (6) describe the orientation dependence of the transverse relaxation time and decay of the signal for a single nanopore or several pores exactly oriented in the same direction. In real samples, a deviation from the ideal ordering of nanopores is usually observed. Moreover, the volumes and form factors can be different for various nanopores.

Therefore, to compare the theoretical predictions with experimental results, it is necessary to average the obtained expression (6) for $S(t,\theta_{S},\zeta ,\xi )$ over all possible orientations of nanopores and their volumes and form factors. Since the distribution of the pore orientation is not known primordially, we assumed that the distribution is done by the binary Gaussian function of the polar and azimuthal angles, which yielded good agreement between the experimental data and the theoretical results for various samples [14-22], and that the angular distributions of all types of pores in a sample are the same. Replacing pore volumes and form factors by their effective values, we obtain the normalized signal averaged over the whole sample:

(7) $$\langle S(t,\theta_{S})\rangle =\frac{1}{\langle {S(t,\theta_{S})|}_{t=0}\rangle }\int_{0}^{2\pi }d\xi \int_{0}^{\pi }d\zeta \;\sin\;\zeta \Phi (\zeta ,\xi )S(t,\theta_{S},\zeta ,\xi ),$$

where Φ(ζ,ξ) is the density function that is assumed to be described by a binary Gaussian function:

(8) $$\Phi (\zeta ,\xi )=\exp\left\{-\frac{{\left(\zeta -\zeta_{0}\right)}^{2}}{2\sigma_{\zeta }^{2}}-\frac{{\left(\xi -\xi_{0}\right)}^{2}}{2\sigma_{\xi }^{2}}\right\},$$

$\sigma_{\zeta }$ and $\sigma_{\xi }$ are the standard deviations of the polar ζ and azimuthal ξ angles, respectively. The standard deviations $\sigma_{\zeta }$ and $\sigma_{\xi }$ characterize the degree of nanopore orientation ordering, and the polar $\zeta_{0}$ and azimuthal $\xi_{0}$ angles determine the averaged orientation of the principal axes of the nanopores with respect to the sample **N** -axis.

## 3. Experimental details

### 3.1 Sample

The studied a-Si:H films were grown at the Laboratoire de Physique des Interfaces. The films of 2 μm thick were deposited at 150 °C by dissociation of pure silane (20 cm^3^/min) at a pressure of 76 mTorr, a radio frequency (RF) power of 2 W, and an interelectrode distance of 22 mm onto the c-Si substrate [34].

Small-angle X-ray scattering (SAXS) and NMR data [29-33, 35-37] show that the studied a-Si:H films contain elongated elliptically shaped nanopores, which are orientated nearly parallel to the film growth direction, as shown schematically in Fig. 1. The nanopores are filled with mobile hydrogen molecules, which is confirmed by NMR measurements [29-33, 35-37]. A sandwich of several films was used to increase the ^1^H signal (Fig.1).

### 3.2 NMR measurements

The ^1^H NMR measurements were carried out at ambient temperature (293±0.5K) using a Tecmag pulsed solid-state NMR spectrometer, a home-built NMR probe and an Oxford superconducting magnet with the magnetic field $B_{0}=8.0$ T corresponding to the ^1^H resonance frequency $\omega_{0}=340.54$ MHz. The echo decay of protons in hydrogen molecules in the a-Si:H films was measured at different angles $\theta_{S}$ between the normal to the film surface and the applied magnetic field (Fig.1). The angles were established within the accuracy of ±1°. The spin-spin relaxation times $T_{2}$ were measured using the Carr-Purcell-Meiboom-Gill (CPMG) pulse sequence [38]. The duration of the π∕2 radiofrequency pulse in the sequences was 1.6 μs and the amplitude of the radio frequency field was of $H_{1}=39$ G, which corresponds to the frequency $\omega_{1}=\gamma H_{1}=166.6$ kHz . All measurements were carried out in a static mode.

In this study, we measured the echo decay for nine sample positions determined by the angles $\theta_{S}=0{}^{\circ}$, 15°, 30°, 40°, 54°, 65°, 74°, 81°, and 90°.

**Fig. 2:**
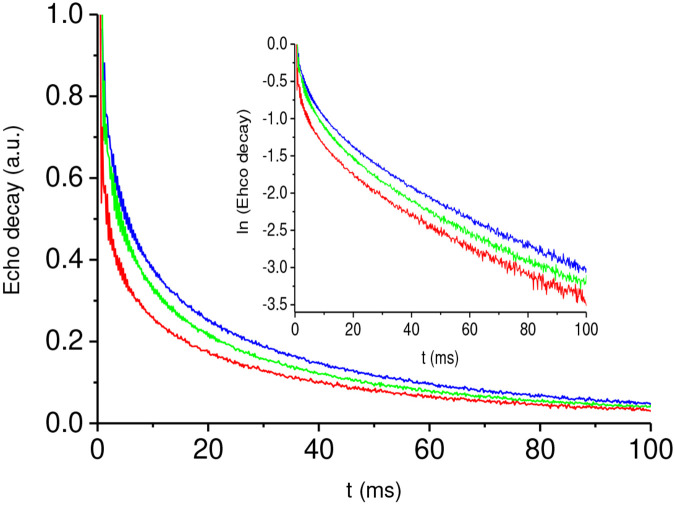
Experimental echo decays, measured using the CPMG pulse sequence and associated with hydrogen molecules, for three angles $\theta_{S}$ between the normal **N** to the film plane and the applied magnetic field, ${\vec{H}}_{0}$ : i) $\theta_{S}=40{}^{\circ}$ blue curve; ii) $\theta_{S}=65{}^{\circ}$ green curve; iii) $\theta_{S}=90{}^{\circ}$ red curve. Inset is the log of the echo decay.

All the decays are similar; some of them are very closed to each other due to nonmonotonic decay dependence on the angle $\theta_{S}$. As an example, the results of our measurements of the echo decay of the a-Si:H films for the angles $\theta_{S}=40{}^{\circ}$, $\theta_{S}=65{}^{\circ}$, and $\theta_{S}=90{}^{\circ}$ are shown in Fig. 2. The results for these angles clear demonstrate the multicomponent character of spin-spin relaxation and its angular dependence.

## 4 Results and Discussion

In our previous papers [20,21] it was assumed that the room-temperature ^1^H NMR signals from a-Si:H films consist of two components associated with (i) hydrogen atoms belonging to Si─H bonds and (ii) $H_{2}$ molecules entrapped in ellipsoid-like nanocavities. Therefore, the echo decays were fitted by a superposition of two exponentials. The longer relaxation time $T_{2}$ was associated with the signal of the $H_{2}$ molecules included in ellipsoid-like nanocavities. The angular dependence of this time was obtained [21, 22] and analysis of this anisotropy allowed one to estimate parameters of the nanostructure [21, 22].

**Fig. 3:**
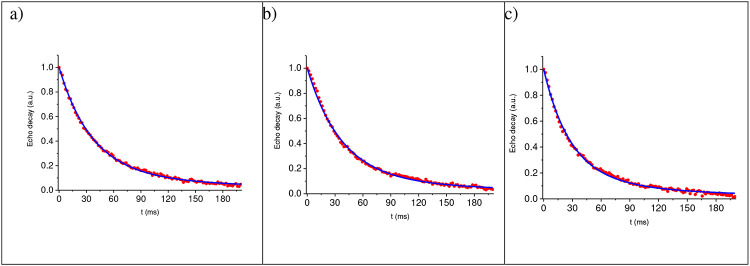
Fitting of the long-time signal decay associated with hydrogen molecules moving in nanopores at various angles $\theta_{S}$ between the normal **N** to the films and the applied magnetic field ${\vec{H}}_{0}$ : a) $\theta_{S}=40{}^{\circ}$; b) $\theta_{S}=65{}^{\circ}$; c) $\theta_{S}=90{}^{\circ}$. Solid red circles are experimental results, solid blue curves are fitting according Eq. (7).

At first, we fitted the experimental data by a two-exponential function, $S_{\mathrm{fit}}(t,\theta_{s})=A_{s}e^{-t∕T_{2s}}+Ae^{-t∕T_{2}}$, using the OriginLab program. The coefficients $A_{s}$ and A depend on the angle $\theta_{S}$ and are close to each other, $A_{s}\approx 0.7\;A$ at all angles. (Note, Figs. 2 and 3 present the normalized decays according to Eq. (7)). However, the characteristic decay times, which are usually considered as the relaxation times, differ by an order of magnitude, $T_{2s}\ll T_{2}$ (Table 1). At *t* > 15−20 ms the signal is practically determined by the long-time decay processes which are characterized by $T_{2}$. This allowed us to separate the slowly decaying signals attributed to mobile hydrogen molecules and further analyze only these processes.

As it is seen from the inset in Fig. 2 done in semi-log scale, the obtained the long-time decays (at *t*> 20 ms) differ from a single exponential function.

### 4.1 Analysis of the relaxation and determination of fitting parameters

A signal associated with hydrogen molecules located inside ellipsoid-like nanocavities was fitted by expression (7). The fitting parameters (η, standard deviations $\sigma_{\zeta }$ and $\sigma_{\xi }$, and averaged angles $\zeta_{0}$ and $\xi_{0}$) were determined by means of the developed computer program based on Piton package, using the non-linear least squares minimization method.

The typical results of fitting of the long-time decays, associated with mobile hydrogen molecules, and the corresponding fitting parameters are presented in Fig. 3 and in Table 2, respectively.

For other angles the obtained parameters were close to the values given in Table 2, spread within ±0.01 for η, ±0.1 for $\sigma_{\zeta }$, and ±0.15 for $\sigma_{\xi }$. This result is expected, since parameters characterizing nanostructure do not depend on sample rotation. While the averaged angles $\zeta_{0}$ and $\xi_{0}$ demonstrate discrepancies (Table 2). The reason for this may be that the relaxation rates in a single nanocavity possess angle symmetry in 3D space [18]. Another reason for the low accuracy in determining these parameters can be relatively weak dependence of the fitting results on the averaged angles. For example, at $\theta_{S}=0$ the function (7) does not depend on $\xi_{0}$ (see Eq. 3). The fitting parameter η and standard deviations $\sigma_{\zeta }$ and $\sigma_{\xi }$ agree well with these parameters obtained previously [21, 22] by analyzing the angle dependence of the relaxation time $T_{2}$. The approach developed here allows us to determine their values using a single time dependence of the signal.

### 4.2 Estimations of the structure parameters of nanopore

Let us estimate the shape and characteristics sizes of the nanopores using the results of fitting (Table 2). From Eq. (5) the ratio $\frac{|F|}{\sqrt{V}}$ is

(10) $$\frac{|F|}{\sqrt{V}}=\frac{4\eta }{\gamma^{2}\hbar \sqrt{3\rho }}.$$

Substituting the determined parameter value n≈0.07 and $\gamma^{2}\hbar =2\pi \times 0.120\frac{{\mathrm{nm}}^{3}}{\mathrm{ms}}$ into Eq. (10) and assuming that according to [30-32] the spin density for $H_{2}$ is $\rho =26\;{\mathrm{nm}}^{-3}$ we obtain,

(11) $$|F|=4.2\times 10^{-2}\sqrt{V}.$$

An experimental study of the nanostructure of a-Si:H films by other methods [27-35] showed that nanopores have an ellipsoidal closed to spherical shape. The method of growing our films was similar to that described by Gericke et al. in [35]. Assuming that the nanopores are spherical, the authors of [35] estimated the diameter of nanopores in the films deposited at a low deposition rate as 1.3 nm. Using this estimation and Eq. (11) we estimate the form factor $|F|=4.7943\times 10^{-2}$. This form factor corresponds to two shapes of the elliptical nanopores [15, Eq. (29)]: i) for F<0 the axis ratio *a/b* = 0.97, the axes *a* = 1.38 nm and *b*= 1.34 nm; ii) for F>0 the axis ratio *a/b*=1.028 , the axes *a* = 1.36 nm and *b*= 1.32 nm. These results agree well with the previously published ones of the small-angle X-ray scattering and NMR data [29-37].

## 5. CONCLUSION

We studied experimentally and theoretically the multicomponent room-temperature ^1^H NMR echo decays in a-Si:H films. The anisotropy of the spin-spin relaxation of protons in H_2_ molecules entrapped in nanopores of these films was experimentally observed and theoretically explained. Despite the multicomponent signal decays, one of the fitting parameters, η, which is related to the size and shape of the nanocavities, is determined quite accurately. This made it possible to determine the size and shape of nanopores in a-Si:H films; our estimations are in good agreement with the results obtained by other methods [33,35]. Some characteristics of the film nanostructure were also determined with an acceptable accuracy. The experimental results are interpreted within the framework of the previously developed theory [14-22] in which a nanoporous material is represented as a set of nanopores containing liquid or gas and the relaxation rate is determined by the dipole-dipole spin interaction, taking into account the restriction of liquid or gas molecule moving inside the pores. Previously, the parameters and nanostructure characteristics such as averaged volume of the pores and their orientation distribution were determined from the angle dependencies of the spin-spin and spin-lattice relaxation times [21,22].

The main difference of the proposed approach is that the above-mentioned parameters can be obtained using a single signal. The method developed by us for the analysis of experimental NMR data is not based on analysis of anisotropy of the relaxation time $T_{2}$, the determination of which is a rather complicated problem in the case of multicomponent decays.

Good agreement between the estimated pore sizes and the results obtained by other methods was achieved by determining several fitting parameters in expression (7). The fitting parameters also provide information about the nanostructure of the sample, such as the standard deviations for the angular distribution of pores, as well as the polar and azimuth angles of the average direction of the pore axes relative to the sample axis. The approach allows one to use the NMR data for quantitative non-destructive determination of the structural parameters of nanoporous materials.

## Figures and Tables

**Table 1:** The typical short ($T_{2s}$) and long ($T_{2}$) relaxation times.

$\theta_{S}$	40°	65°	90°
$T_{2s}$ (ms)	4.97	4.67577	2.7
$T_{2}$ (ms)	44.91	43.87191	38.58

**Table 2:** Fitting parameters at various angles $\theta_{S}$

Angles $\theta_{S}$	0°	15°	30°	40°	54°	65°	74°	*8*1°	90°
$\eta =\frac{\gamma^{2}\hbar }{4}|F|\sqrt{\frac{3\rho }{V}}$, (ms^−1^)	0.08	0.08	0.07	0.07	0.07	0.06	0.07	0.07	0.08
Standard deviation of polar angle $\sigma_{\zeta }$, (rad)	0.3	0.4	0.4	0.4	0.5	0.4	0.4	0.3	0.5
Standard deviation of azimuthal angle $\sigma_{\xi }$, (rad)	0.3	0.4	0.4	0.4	0.5	0.3	0.6	0.6	0.5
Averaged polar angle $\zeta_{0}$, (rad)	0.4	1.5	1.0	0.8	0.6	0.5	0.4	0.3	0.4
Averaged azimuthal angle $\xi_{0}$, (rad)	0.6	1.4	0.7	0.6	0.9	0.8	0.7	1.0	1.2

## References

[1] Abragam A., The Principles of Nuclear Magnetism, Oxford Clarendon Press, 1961.

[2] Callaghan P. T., Principles of Nuclear Magnetic Resonance Microscopy, Oxford Clarendon Press, 1991.

[3] Mehring M., Principles of High Resolution NMR in Solids, 2^nd^ ed., Springer, Berlin, 1983.

[4] Blumich B., NMR Imaging of Materials, Oxford Clarendon Press, 2000

[5] Breitmaier E., Structure Elucidation by NMR in Organic Chemistry: A Practical Guide, John Wiley&Sons Ltd., 2002.

[6] Macomber R.S., A Complete Introduction to Modern NMR Spectroscopy, Wiley, Chichester, 1998.

[7] Hausser K. H., Kalbitzer H. R., NMR in Medicine and Biology: Structure Determination, Tomography, In Vivo Spectroscopy (Physics in Life Sciences), Springer-Verlag Berlin Heidelberg, 1991.

[8] Xia Y., Momot K., Biophysics and biochemistry of cartilage by NMR and MRI, The Royal Society of Chemistry, Cambridge UK, 2016

[9] Panich A. M., NMR Study of the F─H…F Hydrogen Bond. Relation Between Hydrogen Atom Position and F─H…F Bond Length. Chemical Physics 196, 511–519 (1995).

[10] Thommes M., Schlumberger C., Characterization of Nanoporous Materials, Annu. Rev. Chem. Biomol. Eng. 12, 137–62 (2021). 33770464 10.1146/annurev-chembioeng-061720-081242

[11] Mohebbi B., Claussen J., Blümich B., Fast and robust quantification of liquid inside thin fibrous porous materials with single-sided NMR. Magnetic Resonance Imaging. 56: 131–137 (2019). DOI: 10.1016/J.Mri.2018.09.022 ) 30269952

[12] Panich A. M., Nuclear magnetic resonance study of fluorine–graphite intercalation compounds and graphite fluorides, Synthetic Metals 100, 169–185 (1999).

[13] Panich A.M., Belitskii I.A., Moroz N.K., Gabuda S.P., Drebushchak V.A., Seretkin Y.V., Ionic and Molecular Diffusion and Order-Disorder Phase Transition in the Thallium Form of Natrolite, Zh. Strukt. Khimii, 31 (1990). 67–73.

[14] Furman G. B., Goren S. D., Meerovich V. M., Sokolovsky V. L., Anisotropy of spin–spin and spin–lattice relaxation times in liquids entrapped in nanocavities: Application to MRI study of biological systems, JMR, 263, 71–78 (2016). 26773529 10.1016/j.jmr.2015.12.015

[15] Furman G. B., Goren S. D., Meerovich V. M., Sokolovsky V. L., Correlation of transverse relaxation time with structure of biological tissue, JMR, 270, 7–11 (2016). 27380185 10.1016/j.jmr.2016.06.018

[16] Furman G. B., Goren S. D., Meerovich V. M., Sokolovsky V. L., Dipole-dipole interactions in liquids entrapped in confined space, Journal of Molecular Liquids 272, 468–473 (2018).

[17] Furman G., Meerovich V., Sokolovsky V., Xia Y., Spin locking in liquid entrapped in nanocavities: Application to study connective tissues, Journal of Magnetic Resonance 299, 66–73 (2019). 30580046 10.1016/j.jmr.2018.12.012 PMC6942517

[18] Furman G., Meerovich V., Sokolovsky V., Xia Y., Spin-lattice relaxation in liquid entrapped in a nanocavity, Journal of Magnetic Resonance 311, 106669 (2020). 31881481 10.1016/j.jmr.2019.106669 PMC8829806

[19] Furman G, Goren S., Meerovich V., Panich A., Sokolovsky V., Xia Y. Anisotropy of transverse and longitudinal relaxations in liquids entrapped in nano- and micro-cavities of a plant stem, Journal of Magnetic Resonance 331, 107051 (2021) 34455368 10.1016/j.jmr.2021.107051 PMC8842490

[20] Furman G., Meerovich V., Sokolovsky V., Xia Y., Salem S., Shavit T., Blumenfeld-Katzir T., Ben-Eliezer N., Determining the internal orientation, degree of ordering, and volume of elongated nanocavities by NMR: Application to studies of plant stem, Journal of Magnetic Resonance 341, 107258 (2022). 35753185 10.1016/j.jmr.2022.107258 PMC9986720

[21] Panich A., Furman G., Sokolovsky V., Xia Y., Roca i Cabarrocas P., Anisotropic Spin–Lattice and Spin–Spin Relaxations in Hydrogen Molecules Trapped in Non-Spherical Nanocavities, Applied Magnetic Resonance 54, 371–381 (2023) 10.1007/s00723-022-01515-6

[22] Furman G., Panich A., Sokolovsky V., Xia Y., Nanostructure of hydrogenated amorphous silicon (a-Si:H) films studied by nuclear magnetic resonance, Journal of Magnetic Resonance 350, 107434 (2023) DOI: 10.1016/j.jmr.2023.107434. 37080070

[23] Xia Y., Magic-Angle Effect in Magnetic Resonance Imaging of Articular Cartilage, Invest. Radiol. 35, 602 (2000). 11041155 10.1097/00004424-200010000-00007

[24] Xia Y., Relaxation anisotropy in cartilage by NMR microscopy (μMRI) at 14-μm resolution, Magn. Reson. Med. 39, 941–949 (1998). 9621918 10.1002/mrm.1910390612

[25] Zheng S.K., Xia Y., Multi-components of T2 relaxation in ex vivo cartilage and tendon, J. Magn. Reson. 198, 188 (2009). 19269868 10.1016/j.jmr.2009.02.005 PMC2680930

[26] Reiter D.A., Lin P.-C., Fishbein K.W., Spencer R.G., Multicomponent T2 relaxation analysis in cartilage, Magn. Reson. Med. 61, 803 (2009). 19189393 10.1002/mrm.21926 PMC2711212

[27] Fel’dman E. B., Furman G. B., and Goren S. D., Spin locking and spin–lattice relaxation in a liquid entrapped in nanosized cavities, Soft Matter, 8, 9200 (2012).

[28] Fel’dman E.B., Rudavets M.G., Nonergodic nuclear depolarization in nanocavities. J. Exp. Theor. Phys. 98, 207–219 (2004). 10.1134/1.1675888

[29] Williamson D.L., Nanostructure of a-Si:H and related materials by small-angle x-ray scattering, Mat. Res. Soc. Symp. Proc. 377, 251–262 (1995)

[30] Xu X., Yang J., Guha S., Hydrogen dilution effects on a-Si:H and a-SiGe:H materials properties and solar cell performance, J. Non-Crystal. Solids 198-200, 60–64 (1996)

[31] Mahan A.H., Xu Y., Beyer W., Perkins J.D., Vanecek M., Gedvilas L.M., Nelson B.P., Structural properties of hot wire a-Si:H films deposited at rates in excess of 100 Å/s, J. Appl. Phys. 90, 5038–5047 (2001)

[32] Chabal Y. J., Patel C. K. N., Molecular hydrogen in a-Si: H, Rev. Mod. Phys. 59, 835 (1987).

[33] Baugh J., Kleinhammes A. , Han D. , Wang Q. and Wu Y. , Confinement effect on dipole-dipole interactions in nanofluids, Science, 294, 1505–1507 (2001). 11711669 10.1126/science.1065373

[34] Roca i Cabarrocas P., Chévrier J.B., Hue J., Lloret A., Parey J.Y., and Schmitt J.P.M., A fully automated hot-wall multiplasma-monochamber reactor for thin film deposition, J. of Vac. Sci. and Technol. A9, 2331 (1991).

[35] Gericke E., Melskens J., Wendt R., Wollgarten M., Hoell A., and Lips K., Quantification of Nanoscale Density Fluctuations in Hydrogenated Amorphous Silicon, Phys. Rev. Lett., 125, 185501 (2020). 33196241 10.1103/PhysRevLett.125.185501

[36] Young D. L., Stradins P., Xu Y., Gedvilas L. M., Iwaniczko E., Yan Y., Branz H. M., Wang Q., Nanostructure evolution in hydrogenated amorphous silicon during hydrogen effusion and crystallization. Appl. Phys. Lett. 90, 081923 (2007).

[37] Carlos W.E., Taylor P.C., Molecular hydrogen in a-Si:H, Phys. Rev. B 25, 1435–1438 (1982)

[38] Meiboom S., Gill D., Modified Spin-Echo Method for Measuring Nuclear Relaxation Times, Rev. Sci. Instrum. 29, 688–691 (1958).

